# Low-Cost Beamforming Concept for the Control of Radiation Patterns of Antenna Arrays Installed onto UAVs

**DOI:** 10.3390/s21134265

**Published:** 2021-06-22

**Authors:** Leonardo C. dos Santos, Edson R. Schlosser, Marcos V. T. Heckler

**Affiliations:** Laboratory of Electromagnetics, Microwaves and Antennas—LEMA, Federal University of Pampa—UNIPAMPA, Alegrete 97546-550, Brazil; leonardosantos.aluno@unipampa.edu.br (L.C.d.S.); marcosheckler@unipampa.edu.br (M.V.T.H.)

**Keywords:** unmanned aerial vehicles, installed performance, beamforming, antenna arrays, pattern synthesis

## Abstract

This paper presents a low-cost architecture that allows for beamforming with antenna arrays installed onto unmanned aerial vehicles (UAVs). Beam switching is proposed to improve the antenna gain towards the ground station with two three-element arrays installed below the wings of the UAV. The electromagnetic modeling of the complete structure (UAV and integrated antennas) was performed with commercial electromagnetic simulator Ansys HFSS. The radiation patterns were synthesized with particle swarm optimization (PSO). By employing lumped surface-mount device (SMD) components and switches, the design of the feeder to deliver proper excitation coefficients to the antennas is presented, and its performance was assessed by simulations. The proposed approach is demonstrated to be very effective with low-cost production.

## 1. Introduction

Unmanned aerial vehicles (UAVs) are an emerging technology. There are several applications and conventional technologies with lower costs where UAVs can perform. One example is in agriculture, where UAVs are being used for aerial spraying. Besides the lower costs involved, UAVs can fly safely at lower heights than ordinary aircraft can, and this is an important trait to minimize the drift of the poison or fertilizer being sprayed. Other important applications are environmental monitoring or disaster management. In these cases, large areas can be surveyed without exposing human beings to hazardous environments.

In order to improve safety, sophisticated systems are integrated into aircraft for communication and navigation purposes [1]. In [2,3,4,5,6], antennas for installation onto UAVs were proposed. However, the influence of the UAV structure on the radiation properties of these antennas has not been analyzed. This is an important issue to be taken into account for aircraft-antenna design, especially if new positions for antenna installation are envisaged, such as in the wings [7,8] and in the horizontal stabilizers [9].

According to civil aviation regulations, it is mandatory to assess the radiation properties of antennas installed onto airplanes. A classical way to assess the installed performance of antennas is by inflight measurements [10]. The main drawbacks are the costs involved and the difficulty to precisely obtain radiation pattern cuts. This is only possible after proper synchronization between the positions of the aircraft, sampled during the measurements, with pattern-measured data. Although possible, it is not easy to perform this procedure is not easy for full-scale aircraft. In the case of UAVs, since dimensions are smaller, on-ground tests are possible by installing it on a positioner in an anechoic chamber.

Another way to assess the radiation properties of antennas installed onto aircraft is by means of electromagnetic simulations. In [11], a comparison between simulations and on-ground measurements of a full-scale aircraft was presented, whereby good agreement between numerical predictions and measured data could be verified. Measurements with scaled models, i.e., mock-ups of aircraft with reduced dimensions, were compared with simulations, and good agreement was again obtained [12,13,14,15]. Hence, good engineering predictions can be obtained with simulations provided that appropriate modeling is carried out.

In the above references, analyses considered that airframes are made of perfect electric conductors. This assumption is appropriate for most commercial aircraft. However, UAVs are built with parts made of plastic, wood, and other nonconductive materials. Therefore, another approach for simulations needs to be taken into account. Analysis of a UAV composed of dielectric materials was presented in [16], where antenna arrays were designed to yield nearly uniform coverage in the yaw plane. Other recent contributions of the installed performance of antennas on aerial vehicles are presented in [17,18].

In this paper, a new architecture to use switchable beams is proposed. Although the same UAV is considered as in [16], operation frequency was reduced in order to reduce free-space loss, hence increasing the flight range from the controlling ground station. Additionally, the concept shown in this paper allows for switching the main beam depending on the relative location of the UAV from the ground station, so as to improve the operation gain of the antennas on the UAV in comparison to [16]. The main contribution of this work is to show that the proposed beamforming architecture is simple to implement and effective for the beam switching of arrays installed onto UAVs.

The paper is organized as follows: Section 2 presents the UAV, antenna array geometry, and the simulation and design approach. Obtained results are presented in Section 3. Lastly, results are discussed in Section 4.

## 2. Materials and Methods

For aerial vehicles, smaller antennas have less impact on aerodynamics, so higher frequencies may be preferred, especially for the integration of antennas onto UAVs. Therefore, the present study was carried out at 2.4 GHz, which is also an ISM frequency and unlicensed in many countries.

### 2.1. Electromagnetic Modeling of UAV and Antenna Arrays

**Figure 1:**
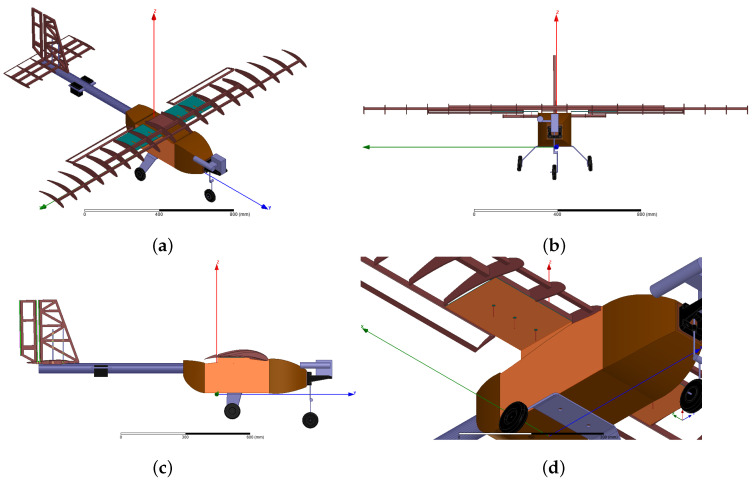
Unmanned aerial vehicle (UAV) geometry: (**a**) General view; (**b**) Front view; (**c**) Side view; (**d**) Antenna array installed below right wing.

The electromagnetic modeling of the UAV considered in this study is presented in Figure 1. It was mainly composed of wood (shown in brown) with a few metallic parts (shown in gray). In the UAV prototype, the wings and rear stabilizers were covered by a thin plastic film. Since this film was electrically very thin compared to the wavelength at 2.4 GHz, it should not strongly impact the electromagnetic properties of the UAV; hence, it was removed for electromagnetic simulations. The engine was located at the very front part of the UAV, and the propeller was removed. For simulations, the coordinate system was positioned so that the wingspan was located along the *x* axis ($\phi =0^{\circ }$), while aircraft length was along the *y* axis. The *z* axis pointed vertically to the zenith ($\theta =0^{\circ }$). For analyses that follow, the roll plane corresponds to the xz plane ($\phi =0^{\circ }$), the pitch plane to the yz plane ($\phi =90^{\circ }$), and the yaw plane to the xy plane ($\theta =90^{\circ }$). Wingspan was 1.868 m and length was 1.467 m. At 2.4 GHz, these dimensions correspond to 14.9 $\lambda_{0}$ and 11.7 $\lambda_{0}$, respectively, where $\lambda_{0}$ is the wavelength in free space; hence, the UAV had a moderate size in comparison to operating wavelength.

Communication with the ground station was achieved with two independent arrays installed below the wings. Figure 1d presents the array geometry below the right wing (the same geometry holds for the array installed below the left wing). Orange stands for surfaces covered with copper, so as to act as ground plane and lateral reflector. The ground plane had dimensions of 150 × 225 mm, which, in wavelengths, corresponds to 1.2 $\lambda_{0}$ × 1.8 $\lambda_{0}$. The lateral reflector had dimensions of 350 × 161 mm, which equates to 2.8 $\lambda_{0}$ × 1.3 $\lambda_{0}$. The distance between adjacent antennas was $\lambda_{0}/2$, and the distance of the first antenna from the lateral conducting wall was $\lambda_{0}/4$. The antennas that composed the arrays were standard quarter-wave monopoles, which were fed by microstrip lines printed on a laminate located above the ground plane.

### 2.2. Proposed Beamforming Architecture

In order to reduce complexity, arrays were steered so as to allow for operation under three beamforming conditions. The first configuration, labeled as Case 1, corresponds to the generation of beams to the front part of the aircraft. The second configuration, labeled as Case 2, was used to generate a beam in a direction between the *x* and *y* axis. Case 3 generates a single beam per array along the wings.

**Figure 2:**
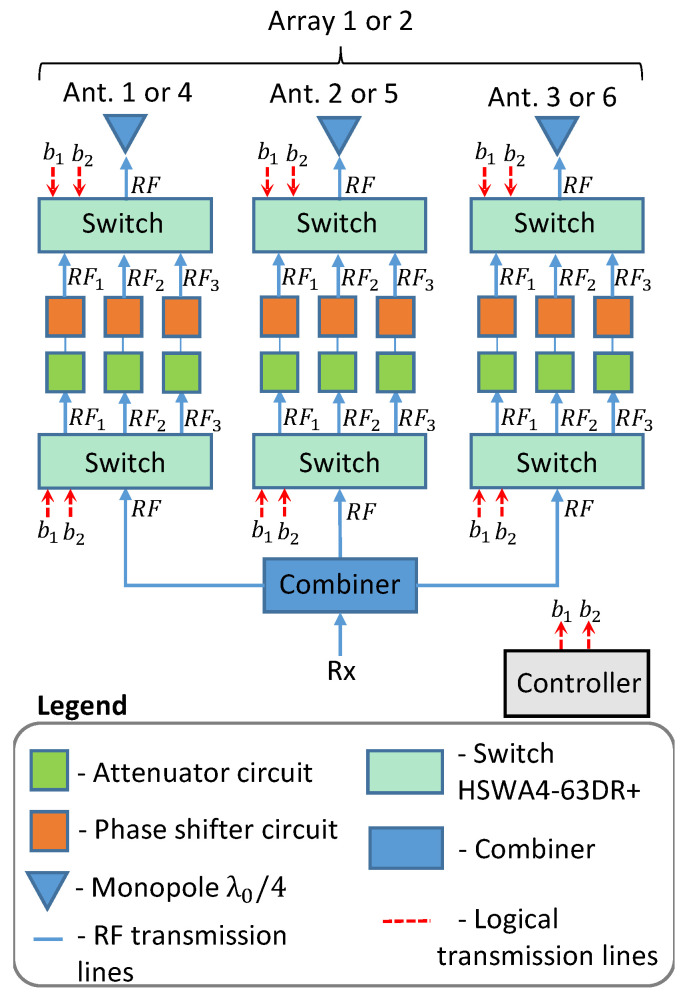
Block diagram of proposed beamforming architecture.

In order to achieve these beamforming conditions with a feeder on board the UAV, a simple and compact architecture is proposed in this paper. The block diagram is depicted in Figure 2. Each radio-frequency (RF) channel ($RF_{1}$, $RF_{2}$, or $RF_{3}$) was composed of a pair of RF switches that were used to choose the beamforming scenario (Cases 1, 2, or 3) that needed to be established, one passive attenuator and a passive phase shifter. Only two bits were needed to drive all switches according to the truth table shown in Table 1. The controller needs to compare the position of the UAV with that of the ground station to choose the array to be used (array 1 under the right wing or array 2 under the left wing) and the appropriate beamforming case.

In order to yield a low-cost solution, attenuators and phase shifters were designed using lumped elements in the surface-mounted device (SMD) format. Attenuators were designed using standard Π sections of resistors that had been calculated for the desired attenuation [19]. Phase shifters were designed with Π and T sections composed of inductors and capacitors depending on whether a positive or negative phase shift is desired. The values of inductance and capacitance were calculated by considering the equivalent circuit of a transmission line with electrical length corresponding to the desired phase shift [20]. The resulting design of these RF channels is presented in Section 3.

### 2.3. Performance of Antennas Installed onto UAV

**Figure 3:**
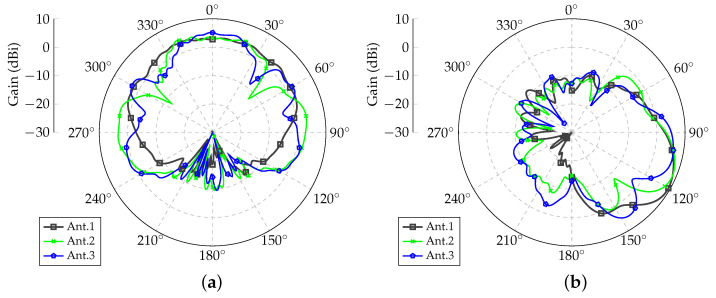
Embedded radiation patterns for Array 1 (below right wing). (**a**) Azimuth planes for $\theta =90^{\circ }$; (**b**) Elevation planes for $\phi =0^{\circ }$.

**Figure 4:**
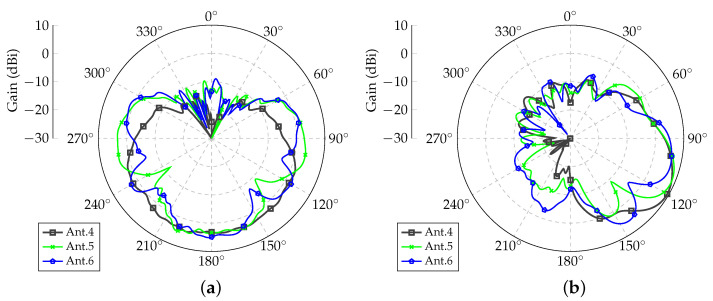
Embedded radiation patterns for Array 2 (below left wing). (**a**) Azimuth planes for $\theta =90^{\circ }$; (**b**) Elevation planes for $\phi =180^{\circ }$.

In order to verify the performance of each monopole, a preliminary simulation was run in Ansys HFSS electromagnetic simulator. Computations were set up using a combination of the finite-element and the boundary integral (FEBI) methods. After the mesh optimization process, the complete structure was modeled with 410,913 tetrahedra. For this analysis, only one antenna was fed at a time while terminating the remaining monopoles with matched loads. The resulting radiation patterns are shown in Figure 3 and Figure 4. Antennas 1 and 4 were the ones closest to the lateral wall of the airframe (see Figure 1d), Antennas 3 and 6 were the farthest from the airframe, and Monopoles 2 and 5 were the center elements of Arrays 1 and 2, respectively. Each antenna had its own embedded radiation pattern; hence, the assumption that all elements radiate equally, as normally performed in classical array theory, is not valid in the present case due to the influence of the UAV parts on antenna characteristics. The embedded patterns of both arrays were nearly mirrored with only very small differences that could be attributed to the precision of the numerical technique used by the electromagnetic simulator.

**Figure 5:**
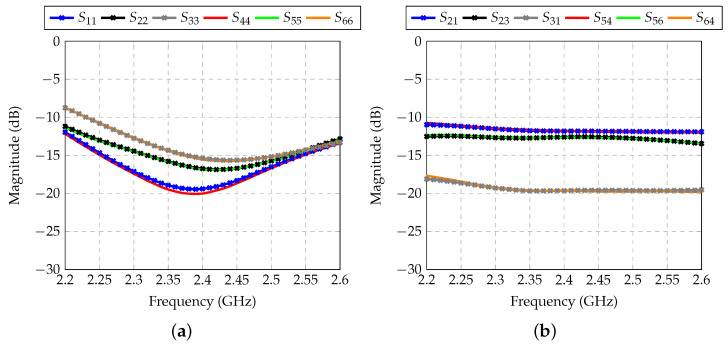

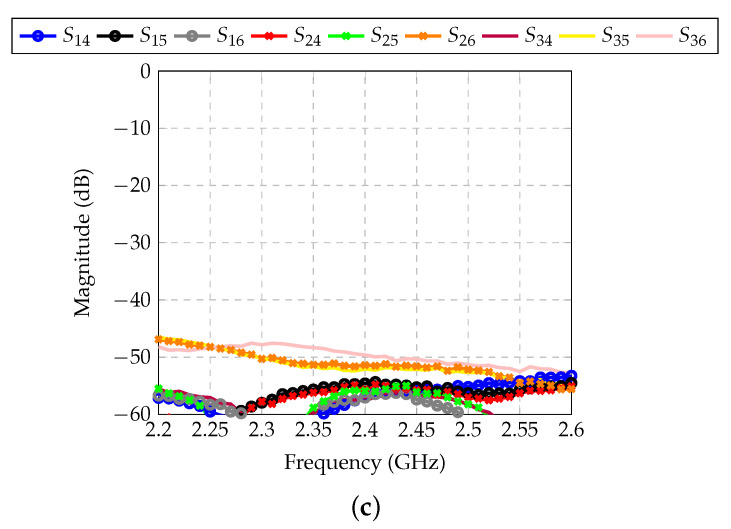
S-parameters of the antenna arrays: (**a**) Reflection at antenna inputs; (**b**) Mutual coupling between antennas inside each array; (**c**) Mutual coupling between antennas of different arrays.

The effect of impedance matching and mutual coupling was also assessed by analyzing the scattering parameters (S parameters) of the arrays. Curves as a function of frequency are shown in Figure 5. The level of reflected power at the input of each antenna was below −10 dB nearly in the whole band of analysis (2.2–2.6 GHz), and was below −15 dB in the central frequency (2.4 GHz). This means that the impedance matching of each monopole was satisfactory. Mutual coupling was moderate between elements in the same array (Figure 5b), while the antennas of Array 1 were highly isolated from the antennas of Array 2 (Figure 5c). Hence, beamforming analyses did not need to be carried out by simultaneously exciting the six antennas, and the arrays could be independently treated.

### 2.4. Application of Particle Swarm Optimization to Arrays Installed onto UAV

Radiation pattern synthesis was widely reported in the literature. In simplified formulations, isotropic antennas are considered [21,22]. In the case of antennas installed onto a UAV, since the embedded pattern of each monopole is different, the optimization of the radiation characteristics of the arrays is only possible by using complete formulation and with an optimization technique. Among other methods available in the literature, particle swarm optimization (PSO) is widely used to solve electromagnetic and antenna problems [23,24,25]. PSO is based on swarm dynamics, whereby individuals (or particles) composing the swarm stand for potential solutions for an optimization problem. In the present case, a particle is a vector containing magnitudes and phases to be imposed at the terminals of each antenna. As verified in the preceding section, arrays can be independently treated, so that the *n*-th particle can be represented mathematically by

(1) $$p_{n}=\left[\begin{array}{cccccc} a_{g} & \delta_{g} & a_{g+1} & \delta_{g+1} & a_{g+2} & \delta_{g+2} \end{array}\right],$$

with g=1 for Array 1, and g=4 for Array 2, where terms $a_{i}$ and $\delta_{i}$ stand for the magnitude and phase of the *i*-th excitation coefficient, respectively, to be imposed at the terminals of the *i*-th antenna.

**Figure 6:**
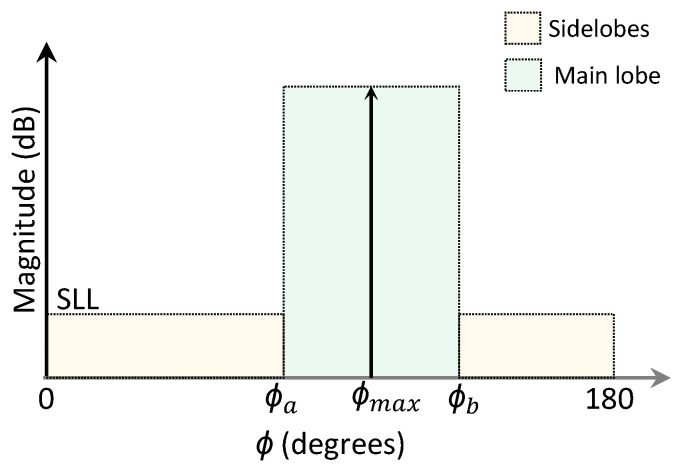
Mask for desired radiation patterns.

The ability of a particle to solve the optimization problem is measured by a fitness function that is based on the error between the desired and the calculated radiation pattern according to [26]. A symmetry plane was used in the optimization process due to the characteristic of the monopole radiation pattern and the use of metallic reflectors on the sides of the UAV, so the evaluation of a fitness function was realized considering the variation of ϕ between 0${}^{\circ }$ to 180${}^{\circ }$ for Arrays 1 and 2. The desired radiation pattern is represented by a mask, which is illustrated in Figure 6, where $\phi_{max}$ is the direction of maximal radiation, and $\phi_{a}$ and $\phi_{b}$ represent the transition angles from the region of the sidelobes to the main lobe.

Optimization was performed considering sidelobe level SLL≤−15 dB and

Case 1: $\phi_{a}$ = 70${}^{\circ }$, $\phi_{max}$ = 85${}^{\circ }$ and $\phi_{b}$ = 120${}^{\circ }$ for Array 1, and $\phi_{a}$ = 80${}^{\circ }$, $\phi_{max}$ = 95${}^{\circ }$ and $\phi_{b}$ = 130${}^{\circ }$ for Array 2;

Case 2: $\phi_{a}$ = 35${}^{\circ }$, $\phi_{max}$ = 60${}^{\circ }$ and $\phi_{b}$ = 100${}^{\circ }$ for Array 1, and $\phi_{a}$ = 95${}^{\circ }$, $\phi_{max}$ = 120${}^{\circ }$ and $\phi_{b}$ = 160${}^{\circ }$ for Array 2;

Case 3: $\phi_{a}$ = $\phi_{max}$ = 0${}^{\circ }$ and $\phi_{b}$ = 50${}^{\circ }$ for Array 1, and $\phi_{a}$ = 130${}^{\circ }$ and $\phi_{b}$ = $\phi_{max}$ = 180${}^{\circ }$ for Array 2.

According to the formulation presented in [24], the PSO was set up with the following parameters:

- Swarm size: 30 particles;
- Maximal number of iterations: 35;
- Inertia (ω): 0.7;
- Self-confidence coefficient ($C_{2}$): 0.5;
- Confidence in the swarm ($C_{1}$): 0.5.

## 3. Results

After the definitions described in Section 2, radiation patterns were optimized considering the individual embedded patterns shown in Figure 3a and Figure 4b.

### 3.1. Optimized Radiation Patterns—Ideal Case

As stated before, the main idea to keep the communication link between the UAV and the ground station is by beam switching. For each array, three beamforming scenarios (cases) were considered, where each case stands for one or two directions where the main beam (or main beams) are to be indicated. The PSO was used to optimize the excitation coefficients so as to point the main beam to the following directions:

For array 1:

- Case 1: the main beam should be pointed towards the UAV front part or $\phi_{max}=85^{\circ }$;
- Case 2: the main beam should be pointed to $\phi_{max}=60^{\circ }$;
- Case 3: the main beam should be pointed towards the right wing or $\phi_{max}=0^{\circ }$.

For array 2:

- Case 1: the main beam should be pointed towards the UAV front part or $\phi_{max}=95^{\circ }$;
- Case 2: the main beam should be pointed to $\phi_{max}=120^{\circ }$;
- Case 3: the main beam should be pointed towards the left wing or $\phi_{max}=180^{\circ }$.

**Figure 7:**
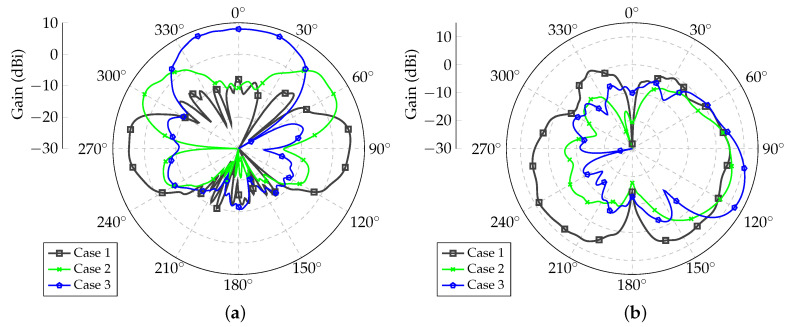
Synthesized radiation patterns for Array 1. (**a**) Yaw plane ($\theta =90^{\circ }$); (**b**) Elevation planes for $\phi =85^{\circ }$ (Case 1), $\phi =60^{\circ }$ (Case 2), and $\phi =0^{\circ }$ (Case 3).

After running PSO, the optimized excitation coefficients are listed in Table 2 for the three beamforming cases listed above. Due to the symmetry of the UAV model and the array’s geometry, the excitation coefficients for Antennas 1 and 4, 2 and 5, and 3 and 6 were the same. The resulting radiation patterns are shown in Figure 7 for Array 1. In the yaw plane, one beam existed at $\phi_{max}=85^{\circ }$, as desired, but another beam at $\phi =275^{\circ }$ also arose. Similarly, for Case 2, one beam existed in the desired direction $\phi_{max}=60^{\circ }$, and another at $\phi =300^{\circ }$ was present. This behavior occurred because of the linear configuration of the arrays considered in this study. For Case 3, however, only one main beam was obtained. In Figure 7b, the patterns produced for the three beamforming scenarios are presented in the elevation planes at $\phi =85^{\circ }$ for Case 1, $\phi \;=\;60^{\circ }$ for Case 2, and $\phi =0^{\circ }$ for Case 3.

**Figure 8:**
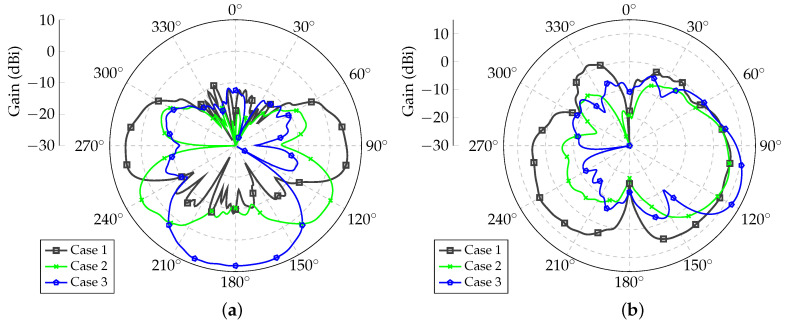
Synthesized radiation patterns for Array 2. (**a**) Yaw plane ($\theta =90^{\circ }$); (**b**) Elevation planes for $\phi =95^{\circ }$ (Case 1), $\phi =120^{\circ }$ (Case 2) and $\phi =180^{\circ }$ (Case 3).

The radiation patterns for Array 2 are shown in Figure 8. It was verified that the pattern shapes were very similar to those obtained for Array 1 but with mirrored behavior.

### 3.2. Design of Feeder for Beamforming

To impose the beamforming coefficients at each antenna terminal, the proper hardware needs to be designed to suit the beamforming strategy. In the present case, the goal was to produce switched beams with low-cost hardware. One of the possibilities was to digitally produce the beamforming coefficients with a digital-processing unit, which demands the use of digital-to-analog converters. The drawback of such an approach is the need to generate baseband signals with proper magnitudes and phases, which need to be upconverted to the RF operating frequency. This demands electronic circuitry along with a power divider to deliver the local-oscillator signal to the upconverting mixers. Hence, the needed hardware may be complex for integration into a UAV. Another strategy is to use digitally controlled variable gain amplifiers and phase shifters. The main drawback is the cost of these components.

**Figure 9:**
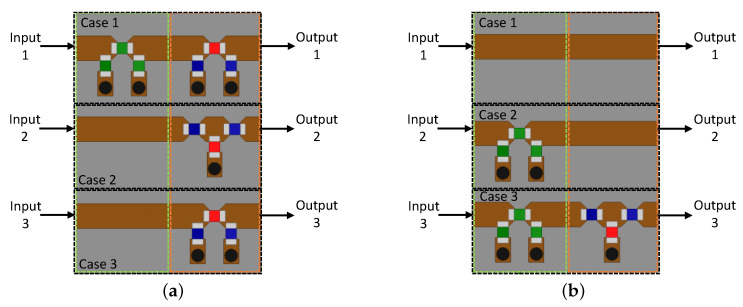

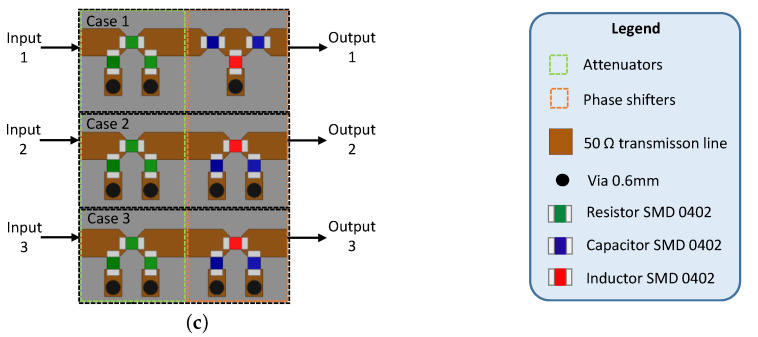
Feeders composed of attenuator and phase shifter units: (**a**) Feeder for Antennas 1 and 4; (**b**) Feeder for Antennas 2 and 5; (**c**) Feeder for Antennas 3 and 6.

The two approaches described above allow for beamforming with large flexibility. However, in the proposed architecture, for the synthesis of only three beamforming conditions, a much simpler feeder can be designed. The attenuators and phase-shifting units can be produced of passive components, yielding a cost-effective solution. As shown in Figure 2, three RF channels are needed for each antenna. The feeder for Antennas 1 and 4 is depicted in Figure 9a. It was composed of three RF channels with one attenuator (enclosed by dashed green line) and one phase shifter (enclosed by dashed orange line). By observing Table 2, there was only the need for an attenuator for Case 1.

The attenuator topology was composed of three SMD resistors with 0402 dimensions in a Π arrangement. The phase-shifter units were composed of SMD inductors and capacitors, also with 0402 dimensions. Only one RF channel was supposed to be used at a time, depending on the needed beamforming situation. This choice is made by the pair of RF switches present in Figure 2. Similarly, feeders for Antennas 2 and 5, and for Antennas 3 and 6, are presented in Figure 9b,c, respectively. All beamforming coefficients listed in Table 2 were synthesized by using the part values given in Table 3, where $R_{s}$, $R_{p}$, $C_{s}$, $C_{p}$, $L_{s}$, and $L_{p}$ stand for the resistors, capacitors, and inductors of the circuits, respectively. Indices *s* and *p* denote series or shunt connection, respectively.

The performance of each RF channel was verified by means of simulations with Ansys HFSS. An electrical simulation without considering the geometrical details of the implementation is not enough to obtain the desired performance, as demonstrated in [20], due to the parasitic capacitances introduced by the transitions between the transmission lines and the SMD components. SMD components were modeled in Ansys HFSS by drawing small blocks, with the same size as that of the real components, and by assigning them with the lumped RLC option.

**Figure 10:**
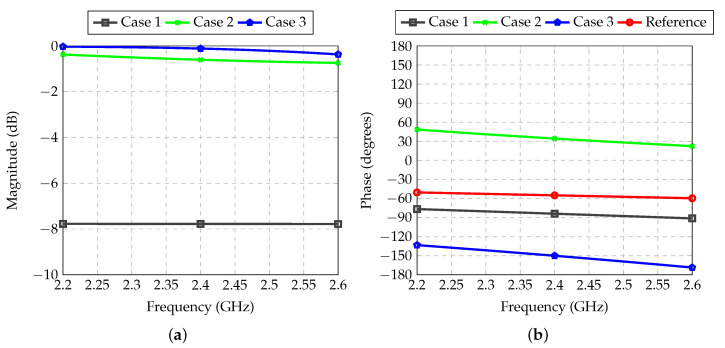
Transmission coefficient between inputs and outputs of each RF path of the feeder for Antennas 1 and 4: (**a**) Magnitude; (**b**) Phase.

The transmission coefficient between inputs and outputs of the feeder for Antennas 1 and 4 is shown in Figure 10. The reference for the phase, shown by the red curve in Figure 10b, is equivalent to the phase shift introduced by a straight 50-Ω transmission line connecting an input to the respective output. This allows for the verification of the actual phase shift introduced by the T or Π sections. For RF channels 2 and 3, no attenuators are needed. The T section used in Channel 2 and the Π section used in Channel 3 introduce insertion losses of 0.6 and 0.1 dB, respectively. In fact, according to Table 2, these two values should ideally be the same, but as is demonstrated below, this unbalance has little impact on the final radiation patterns.

**Figure 11:**
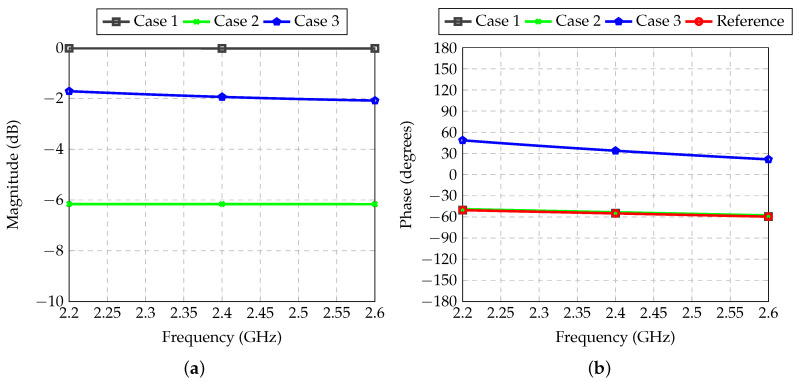
Transmission coefficient between inputs and outputs of each RF path of the feeder for Antennas 2 and 5: (**a**) Magnitude; (**b**) Phase.

The performance of the feeder for Antennas 2 and 5 is demonstrated in Figure 11. In this case, there was only the need to shift the phase in Path 3. Path 1 was the reference channel for the whole system since it did not demand attenuation or phase shift. Therefore, insertion loss was very low (0.03 dB), which was caused only by low losses introduced by the considered microwave laminate, and the phase shift was used as the reference for the whole system.

**Figure 12:**
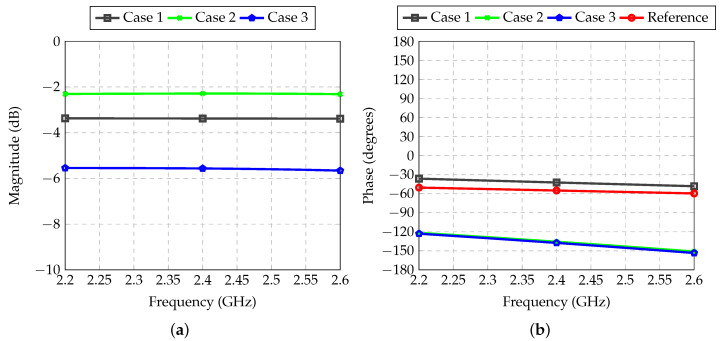
Transmission coefficient between inputs and outputs of each RF path of the feeder for Antennas 3 and 6: (**a**) Magnitude; (**b**) Phase.

The feeder for Antennas 3 and 6 was also analyzed with the electromagnetic simulator. The transmission coefficients are shown in Figure 12. For all designed feeders, the desired values for attenuation and phase shift were obtained.

**Figure 13:**
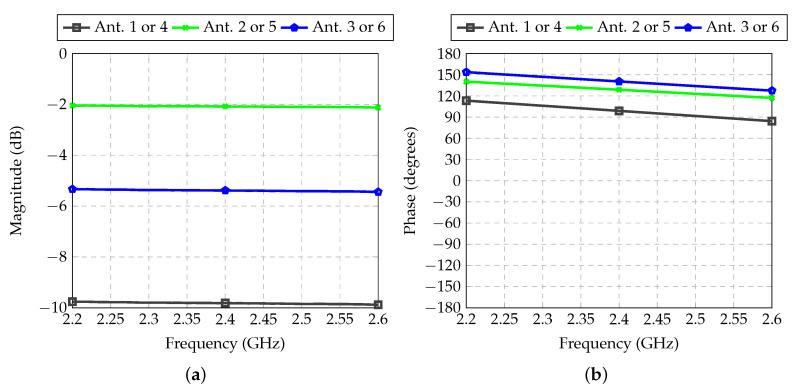
Performance of feeder for Antennas 1 or 4 with inclusion of RF switches: (**a**) Magnitude; (**b**) Phase.

**Figure 14:**
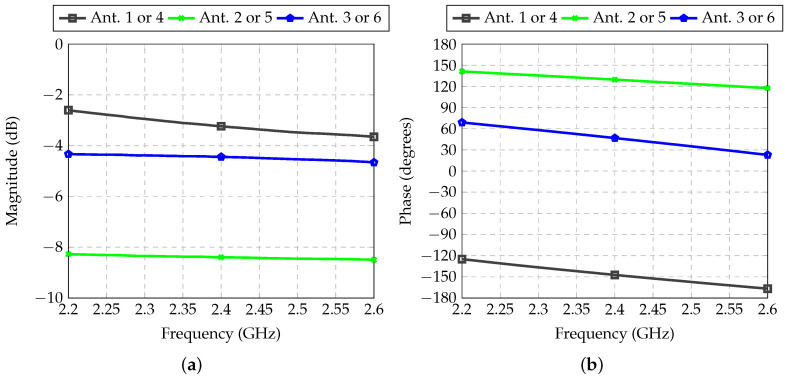
Performance of the feeder for antennas 2 or 5 with the inclusion of the RF switches: (**a**) Magnitude; (**b**) Phase.

**Figure 15:**
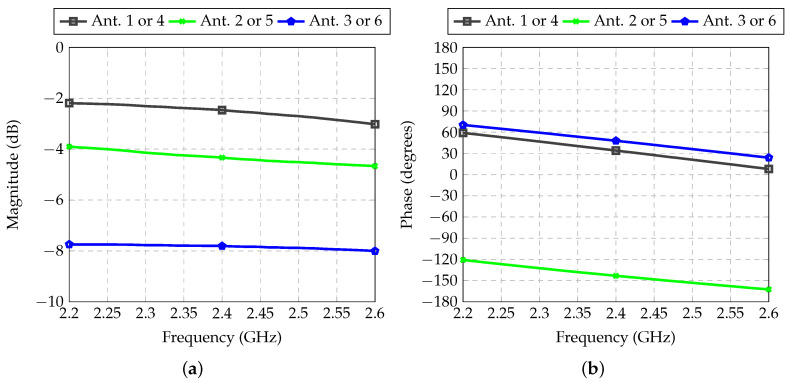
Performance of feeder for Antennas 3 or 6 with inclusion of RF switches: (**a**) Magnitude; (**b**) Phase.

The connection of the RF switches with the attenuators and phase shifters was modeled in cosimulation with Ansys Designer. The switch itself was considered as an S-parameter block, which was set up with the S-parameter data provided by the manufacturer. The chosen model for the present application was HSWA4-63DR+, produced by Mini-Circuits. The performance of the resulting circuits is shown in Figure 13, Figure 14 and Figure 15. The main influence of the switches on the overall performance is the insertion loss, which was verified to be approximately 2 dB.

### 3.3. Final Radiation Patterns

**Figure 16:**
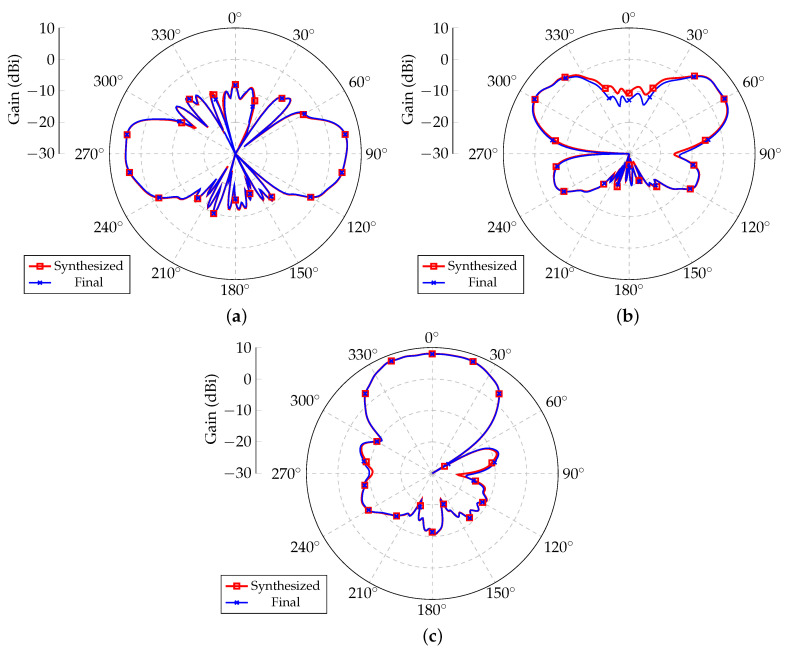
Final radiation patterns for Array 1 in yaw plane ($\theta =90^{\circ }$). (**a**) Case 1: $\phi_{max}=85^{\circ }$; (**b**) Case 2: $\phi_{max}=60^{\circ }$; (**c**) Case 3: $\phi_{max}=0^{\circ }$.

After the complete electromagnetic modeling of the feeder channels, the UAV along with the integrated antennas was simulated again. The excitation coefficients were set to be the values presented in Figure 13, Figure 14 and Figure 15 at 2.4 GHz. Figure 16 shows the resulting patterns in the yaw plane for Array 1. Results indicate that only small deviations were obtained with the produced beamforming coefficients with the designed circuits in comparison to the ideal curves optimized with PSO. With this validation, the proposed approach allows for effective beamforming in the three desired scenarios with a low-cost architecture.

## 4. Discussion

This paper presented an architecture for the beamforming of antenna arrays installed on an unmanned aerial vehicle (UAV). Analysis included extracting the radiation pattern of individual antennas (embedded patterns) in the presence of the UAV structure. As verified in the literature, only few papers dealt with this topic in a systematic approach.

The beamforming of antennas taking their interaction with the UAV airframe is a complicated electromagnetic environment that can only be dealt with an optimization technique, since classical array theory cannot be successfully applied. The application of particle swarm optimization (PSO) allowed for obtaining excitation coefficients that resulted in radiation patterns with beams pointed to specific directions, hence allowing for beam switching. By considering only three beamforming cases, an angular range of nearly 180${}^{\circ }$ could be covered with one antenna array; hence, by installing one array below each wing, the whole yaw plane could be covered with a low pattern ripple.

Simple feeders composed of passive lumped SMD components and RF switches were designed to yield a practical approach for beam switching. A thorough electromagnetic analysis of the feeder was presented, whereby the desired attenuation and phase-shift values had been obtained. Lastly, with excitation coefficients produced with the designed circuitry, the final radiation patterns were very close to the originally synthesized patterns with PSO, with only small deviations visible for beamforming Case 2. These deviations appeared because the phase-shifting sections also introduced additional insertion loss, and the obtained coefficients thereby deviated slightly from the desired ones listed in Table 2. Nevertheless, since the final obtained patterns with the proposed architecture were very close to the ideally synthesized patterns obtained with PSO, the proposed architecture was demonstrated to be very effective, easy to fabricate, and with low production cost.

## Figures and Tables

**Table 1:** Truth table to drive radio-frequency (RF) switches.

Case	Mode ON	$b_{2}$	$b_{1}$
1	RF to $RF_{1}$	Low	High
2	RF to $RF_{2}$	High	Low
3	RF to $RF_{3}$	High	High

**Table 2:** Excitation coefficients obtained with particle swarm optimization (PSO).

Case	Antenna 1 or 4		Antenna 2 or 5		Antenna 3 or 6	
	Atten. (dB)	Phase (deg)	Atten. (dB)	Phase (deg)	Atten. (dB)	Phase (deg)
1	−7.98	−30.37	0	0	−3.48	14.29
2	0	87.93	−6.12	0	−2.26	−81
3	0	−96	−1.93	90	−5.24	−82.4

**Table 3:** Commercial values for calculated surface-mounted device (SMD) 0402 components.

Case	Antenna 1 or 4		Antenna 2 or 5		Antenna 3 or 6	
	Resist. (Ω)	Ind. (nH)	Resist. (Ω)	Ind. (nH)	Resist. (Ω)	Ind. (nH)
		Cap. (pF)		Cap. (pF)		Cap. (pF)
1	$R_{s}=51$	$L_{s}=1.6$	−	−	$R_{s}=20$	$C_{s}=10$
	$R_{p}=120$	$C_{p}=0.35$	−	−	$R_{p}=270$	$L_{p}=13$
2	−	$C_{s}=1$	$R_{s}=39$	−	$R_{s}=13$	$L_{s}=2.7$
	−	$L_{p}=3.3$	$R_{p}=150$	−	$R_{p}=390$	$C_{p}=1$
3	−	$L_{s}=3.2$	$R_{s}=8.2$	$C_{s}=1$	$R_{s}=33$	$L_{s}=2.9$
	−	$C_{p}=1.1$	$R_{p}=620$	$L_{p}=3.2$	$R_{p}=160$	$C_{p}=1$

## Data Availability

Not applicable.

## Abbreviations

The following abbreviations are used in this manuscript:

PSO	Particle swarm optimization
RF	Radio frequency
SLL	Sidelobe level
SMD	Surface-mounted device
UAV	Unmanned aerial vehicle
